# Walking Strategies and Performance Evaluation for Human-Exoskeleton Systems under Admittance Control

**DOI:** 10.3390/s20154346

**Published:** 2020-08-04

**Authors:** Chiawei Liang, Tesheng Hsiao

**Affiliations:** Department of Electrical and Computer Engineering, National Chiao Tung University, Hsinchu 300093, Taiwan; tshsiao@cn.nctu.edu.tw

**Keywords:** exoskeleton, admittance control, ground reaction force, walking strategy, human-exoskeleton system, normalized energy consumption index

## Abstract

Lower-limb exoskeletons as walking assistive devices have been intensively investigated in recent decades. In these studies, intention detection and performance evaluation are important topics. In our previous studies, we proposed a disturbance observer (DOB)-based torque estimation algorithm and an admittance control law to shape the admittance of the human-exoskeleton system (HES) and comply with the user’s walking intention. These algorithms have been experimentally verified under the condition of no ground reaction force (GRF) in our previous studies. In this paper, we devised and integrated with the exoskeleton control system a sensing and communication module on each foot to measure and compensate for GRF. Rigorous theoretical analysis was performed and the sufficient conditions for the robust stability of the closed-loop system were derived. Then, we conducted level ground assistive walking repeatedly with different test subjects and exhaustive combinations of admittance parameters. In addition, we proposed two tractable and physically insightful performance indices called *normalized energy consumption index* (NECI) and *walking distance* in a *fixed period of time* to quantitatively evaluate the performance for different admittance parameters. We also compared the energy consumption for users walking with and without the exoskeleton. The results show that the proposed admittance control law reduces the energy consumption of the user during level ground walking.

## 1. Introduction

In recent years, studies about lower-limb exoskeletons have made significant progress [1,2,3,4,5,6,7,8]. Exoskeletons are assistive or rehabilitative mechatronic devices that actively provide energy to help healthy or gait-disordered people in enhancing or restoring normal locomotion. According to the mobility of the users and desired functionality, the design of exoskeletons may focus on different aspects. In this paper, we aimed at designing control systems of lower-limb exoskeletons that allow healthy people to walk in a more energy-efficient way. Since the target users are those who can walk by themselves, the primary goal of the exoskeleton control system is to comply with the user’s motion. In addition, tractable and physically insightful performance indices for the compliance control system should be identified such that the control parameters can be fine tuned accordingly, and the benefits of the exoskeleton can be quantitatively evaluated and compared.

Various methods for enhancing the compliance of exoskeletons have been developed, including feeding back biological signals of the user for intention detection, reducing the stiffness of the actuators by novel mechanical design, or exploring the delicate compliance control algorithms. Biological signals such as electromyography (EMG) and electroencephalography (EEG) directly reflect the user’s intention; hence, they are very useful for implementing compliance control. The hybrid assistive leg (HAL) in [9,10] measured the user’s EMG as control inputs; Gui et al. [11] proposed an adaptive method to estimate active joint torques by EMG signals. Lyu et al. [12] developed a rehabilitation system for home use that includes an EMG-controlled exoskeleton. The EMG signal can predict the user’s intention since it comes before the contraction and relaxation of muscles; however, it is inconvenient for the user to attach and detach EMG sensors to and from the skin. Another commonly used biological signal is EEG. In [13], Liu et al. developed a brain-controlled lower-limb exoskeleton with two EEG modalities. Lee et al. [14] established a brain–machine interface for the online control of a powered lower-limb exoskeleton based on the EEG signals recorded over the user’s sensorimotor cortical areas. Since the EEG-based methods need to measure and decode EEG signals through a model which is trained for each individual user, the process is time consuming and reliability is not guaranteed.

Another type of compliance control method relies on low-stiffness actuators. In [15], Lv et al. developed a walking assistive exoskeleton with customized backdrivable motors and torque/force sensors, whereas series elastic actuators (SEAs) with admittance/impedance control laws were used to implement the hip-joint exoskeleton in [16] and the knee orthosis in [17]. Although low-stiffness actuators endow the exoskeleton with desirable compliance, designing customized actuators that meet multiple stringent constraints on volume, weight, power consumption, heat dissipation, available output torque, and low stiffness is very challenging.

In addition, the compliance of exoskeletons can be achieved by delicately designed control algorithms. The Berkeley lower extremity exoskeleton (BLEEX) implemented a control algorithm that increases the magnitude of its sensitivity function [18,19,20] with respect to the user’s motion such that the user can drive the exoskeleton with subtle movements; however, robust stability margin may be an issue due to the large gain of the sensitivity function. In [21], Nagarajan et al. developed a linearized-model-based control law which shapes the mechanical admittance of the hip joint to amplify the user’s motion. Since the admittance was determined in advance, it cannot be adjusted online to meet the different requirements for various gaits and walking conditions.

To achieve walking assistance for healthy people, it is crucial for the control law to be highly compliant and highly adaptive with minimal sensor requirements. In our previous work [22,23], we did develop such an admittance control scheme based on estimated user joint torques. The torques exerted by the user to the so-called *human-exoskeleton system* (HES), i.e., the combination of the exoskeleton and the user’s lower limbs, are estimated based on joint angles and motor current without the needs of torque sensors or biological sensors. The estimated user torques indicate the intention of the user, and are used to determine the joint velocity, resulting in a *trajectory-free* admittance control law. Moreover, the admittance is adjustable online, allowing the exoskeleton to adapt itself to different walking conditions. However, the torque estimation algorithm in [23] is susceptible to the interference from the ground reaction force (GRF), and thus all tests in [23] were conducted under the condition of no contact with the ground. To overcome this problem, we extend the work of [23] in this paper by incorporating a sensing and communication module on each foot that measures the GRF and sends it to the central control unit (CCU) to compensate for it. Then, we carry out experiments to verify the effects of different admittance parameters on level ground walking. To quantitatively the compare experimental results, we proposed the *normalized energy consumption index* (NECI) and the *walking distance in a fixed period of time* as the performance indices. The former was calculated based on the estimated user joint torques and represents the normalized energy consumption of the user during walking, while the latter indicates the walking speed of the user with the aid of the exoskeleton. Note that both indices have physical insights and are easy to use since no extra sensors are required to calculate these indices.

To sum up, the contributions of this paper are as follows:

1. A trajectory-free admittance control law for the lower-limbs exoskeletons, which was initiated in our previous work [22,23], were completed and tested. The proposed admittance control law was based on the estimated user joint torques and fully complied with the user’s motion. Furthermore, the admittance of the human-exoskeleton system is adjustable such that the user’s gait can be shaped in a desired way.
2. Tractable and physically insightful performance indices for walking assistive exoskeletons are proposed. According to these performance indices, experimental data for the different subjects and different admittance parameters were evaluated. The results indicate how the admittance parameters quantitatively affect the gait.

We organized this paper as follows. In Section 2, the mechanism of the exoskeleton and the associated electronic modules are described in brief. In addition, the dynamic model of the exoskeleton and the proposed admittance control system structure are introduced. In Section 3, the closed-loop robust stability is analyzed. Then, in Section 4, the experimental results of level ground walking with different test subjects and admittance parameters are presented. Furthermore, we evaluate the effects of the admittance parameters by calculating the performance indices like NECI and walking distance. Finally, the conclusions are made in Section 5.

## 2. Hardware Setting, Models, and System Structure

### 2.1. Hardware Setting

**Figure 1:**
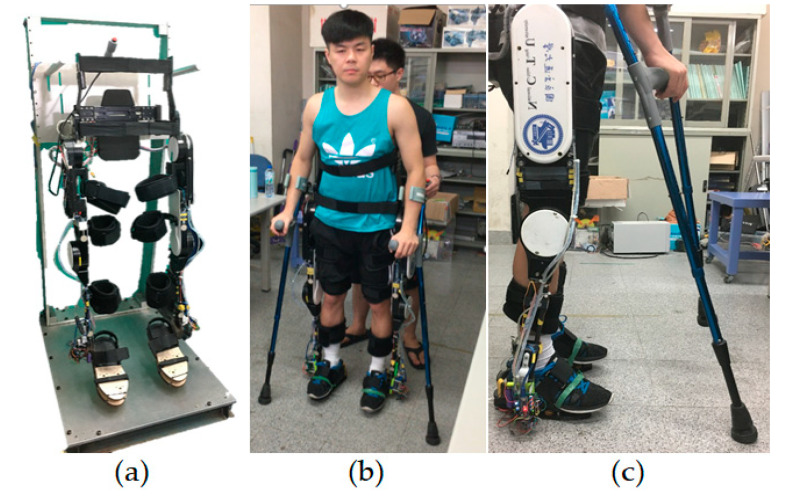
(**a**) Exoskeleton dangles on the rack; (**b**) the front view for a test subject wearing the exoskeleton and standing on the ground; and (**c**) side view for a test subject wearing the exoskeleton and standing on the ground.

The exoskeleton used in this paper was made by the Industrial Technology Research Institute (ITRI), Taiwan (see Figure 1). It is composed of four motors at the hip and knee joints. The original design was for the rehabilitation of patients with complete spinal cord injury. Hence, it was designed to have high stiffness in order to support the weight of the patient. To implement the proposed admittance control law, we modify its hardware and software. We replace the central control unit (CCU) with our own embedded controller (Raspberry Pi), and integrate a sensing, computing and communicating module at each foot to measure and compensate for GRF. Besides, the module also includes a potentiometer mounted next to the ankle to measure the ankle joint angle.

**Figure 2:**
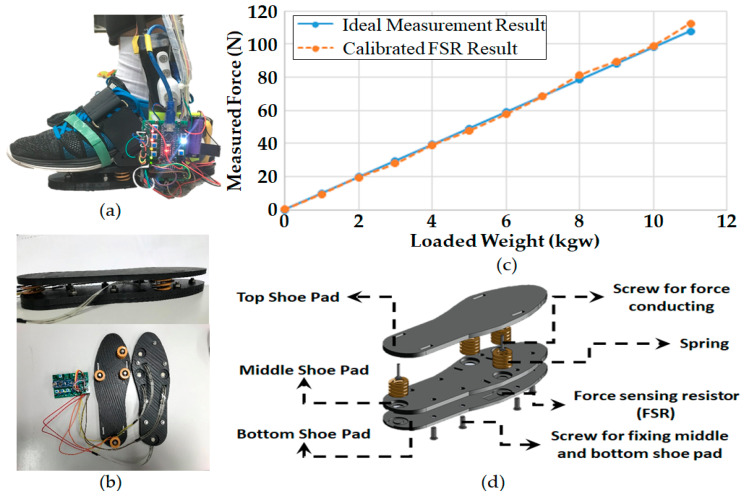
Sensing and communication module on the foot and the calibration result of one FSR; (**a**) Side view of the module installed on the foot; (**b**) photos of the module; (**c**) FSR calibration result. The blue solid line and the orange dashed line represent the ideal and calibrated results, respectively; and (**d**) explosion drawing of the module.

Figure 2a shows the photo of the sensing, computing and communicating module. To get an accurate GRF measurement, an intuitive way is to place as many pressure or force sensors as possible on the sole and sum up all the sensor measurements. However, calibrating numerous sensors is very time-consuming, costly, and therefore intractable. Instead, we place four force sensing resistors (FSRs) between two 3D printed polylactic acid (PLA) shoe pads at the locations, with reference to [24], as shown in Figure 2b,c shows the calibrated measurement of an FSR and compares it with the ideal one. Only the calibration result of one FSR is shown here; however, all FSRs were calibrated individually before they were installed in the sensing module. To distribute the user’s weight on these FSRs, we add the third shoe pad which connects to the second one through four pairs of screws and springs on top of the FSRs (see the explosion drawing in Figure 2d. The screws concentrate the weight on the FSRs while the springs surrounding the screws prevent the FSRs from overloading. The measured data are collected and pre-processed in an Arduino microcontroller. Then, the results are transmitted to the CCU through serial communication channels in real time.

Note that the sensing module is connected to the passive ankle joint of the exoskeleton. When the user puts on the exoskeleton and stands on the ground, the sensing modules on the feet support the weight of the user and the exoskeleton. Thus, the user does not feel the load of the exoskeleton when they are standing, and avoids extra energy consumption from the user due to the weight of the exoskeleton.

### 2.2. Human-Exoskeleton System (HES)

**Figure 3:**
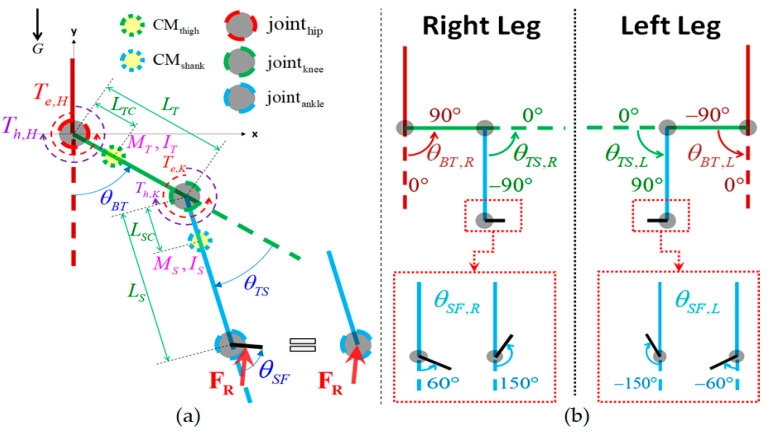
(**a**) Single-leg dynamic model of the exoskeleton; (**b**) the definitions of the joint angle directions and the joint angle limitations. The black, blue, green and brown solid line are the foot, shank, thigh and torso (i.e., body), respectively. The dotted lines represent the limb extension to the connected limb.

The hip and knee joints of the exoskeleton used in this article were actuated by motors on the sagittal plane, while its ankle joints were passive. Since the exoskeleton and the user’s lower limbs are firmly bound together, they are treated as a single system called the *human-exoskeleton system* (HES) in this paper. The HES can also be viewed as the *biomechanical legs* of the user, which is intended to enhance the strength of the user during walking. To simplify the model and the derivation of the model-based torque estimation algorithm, HES is considered as two identical and independent legs, and each leg is modeled as a two-joint planar manipulator with GRF exerting at the ankle as shown in Figure 3a. The positive direction and angular limitations of every joint are defined in Figure 3b. The influence of the internal force between the two hip joints during the stance phase can be diminished by properly tuning the admittance parameters. We will discuss the details in Section 4.2.

The single-leg dynamic model equation is shown as (1):

(1) $$M(\theta )\ddot{\theta }+C(\theta ,\dot{\theta })\dot{\theta }+G(\theta )+B(\dot{\theta })=T_{l}+T_{h}+J^{T}({\left[\theta^{T},\theta_{SF}\right]}^{T})F_{R}\;$$

where $\theta ={\left[\theta_{BT},\theta_{TS}\right]}^{T}$. $\theta_{BT}$ and $\theta_{TS}$ are the hip joint angle and knee joint angle, respectively. $\theta_{SF}$ is the ankle angle. Their directions are defined in Figure 3b. $\dot{\theta }$ and $\ddot{\theta }$ represent the angular velocity and acceleration, respectively. $T_{l}={\left[T_{l,H},T_{l,K}\right]}^{T}$ is the load torque applied from the motors to HES; $T_{h}={\left[T_{h,H},T_{h,K}\right]}^{T}$ is the human (i.e., wearer) input joint torque to HES. Subscripts *H*, *K*, *B*, *T* and *S* denote the quantities associated with the hip, knee, body(torso), thigh and shank, respectively. $F_{R}={\left[F_{R,x},F_{R,y}\right]}^{T}$ is the GRF whereas $J^{T}\left({\left[\theta^{T},\theta_{SF}\right]}^{T}\right)$ is the Jacobian matrix presented as (2). M(θ), $C\left(\theta ,\dot{\theta }\right)$, **G**(θ) and $B\left(\dot{\theta }\right)$ denote the inertia matrix, the Coriolis and centrifugal matrix, the gravitational torque vector and the frictional torque vector, respectively. They are defined as (3).

(2) $$J^{T}({\left[\theta^{T},\theta_{SF}\right]}^{T})=\left[\begin{array}{cc} L_{T}sin(\theta_{TS}+\theta_{SF})+L_{S}sin(\theta_{SF}) & L_{T}cos(\theta_{TS}+\theta_{SF})+L_{S}cos(\theta_{SF})\\ L_{S}sin(\theta_{SF}) & L_{S}cos(\theta_{SF}) \end{array}\right]$$

(3) $$\begin{array}{l} M(\theta )=\left[\begin{array}{cc} I_{T}+I_{S}+M_{T}L_{TC}^{2}+M_{S}(L_{T}^{2}+L_{SC}^{2}+2L_{T}L_{SC}\cos(\theta_{TS})) & I_{S}+M_{S}(L_{SC}^{2}+L_{T}L_{SC}\cos(\theta_{TS}))\\ I_{S}+M_{S}(L_{SC}^{2}+L_{T}L_{SC}\cos(\theta_{TS})) & I_{S}+M_{S}L_{SC}^{2} \end{array}\right]\\ C(\theta ,\;\dot{\theta })=\left[\begin{array}{cc} -M_{S}L_{T}L_{SC}\sin(\theta_{TS}){\dot{\theta }}_{TS} & -M_{S}L_{T}L_{SC}\sin(\theta_{TS})({\dot{\theta }}_{BT}+{\dot{\theta }}_{TS})\\ M_{S}L_{T}L_{SC}\sin(\theta_{TS}){\dot{\theta }}_{BT} & 0 \end{array}\right]\\ G(\theta )=\left[\begin{array}{c} \left(M_{T}L_{TC}+M_{S}L_{T})Gsin(\theta_{BT})+M_{S}L_{SC}Gsin(\theta_{BT}+\theta_{TS}\right)\\ M_{S}L_{SC}Gsin(\theta_{BT}+\theta_{TS}) \end{array}\right]\\ B(\dot{\theta })=\left[\begin{array}{c} B_{BT}{\dot{\theta }}_{BT}+F_{BT}sign({\dot{\theta }}_{BT})\\ B_{TS}{\dot{\theta }}_{TS}+F_{TS}sign({\dot{\theta }}_{TS}) \end{array}\right] \end{array}$$

In the definitions of M(θ), $C\left(\theta ,\dot{\theta }\right)$, **G**(θ) and $B\left(\dot{\theta }\right)$ in (3), *M* is the mass, *L* is the length, and *I* is the moment of inertia. In addition, *G* is the gravity acceleration. *B* and *F* are the viscous and Coulomb friction coefficients, respectively. Subscripts *B*, *T, S*, *C* denote the quantities associated with the body (i.e., torso), thigh, shank, and center of mass, respectively. The parameters in (3) are identified from the experimental data as presented in the previous research [22]. The results are shown in Table 1. As we described in [22], the parameters in Table 1 are identified when there is no user putting on the exoskeleton. These parameters are treated as *nominal parameters* in this paper. Parameter variations due to different users will be explicitly taken into account in the controller design and the closed-loop system analysis in subsequent subsections.

### 2.3. Human-Exoskeleton System Including Motor Servo Control Loop

The dynamic model of the motor servo control loop is presented in this subsection. We consider only one leg here since the two legs are structurally identical. Let $g=\mathrm{diag}\left(g_{H},g_{K}\right)$ be the gear ratio matrix and $\theta_{m}={\left[\theta_{m,H},\theta_{m,K}\right]}^{T}$ be the vector of motor angles. The relationship between the motor angle and the HES joint angle can be represented as (4)

(4) $$\theta_{m}=g\theta$$

The dynamic model of the motor’s rotor can be described as (5)

(5) $$N_{m}{\ddot{\theta }}_{m}+D_{m}{\dot{\theta }}_{m}=T_{m}-g^{-1}T_{l}$$

where $N_{m}=\mathrm{diag}\left(N_{m,H},\;N_{m,K}\right)$ and $D_{m}=\mathrm{diag}\left(D_{m,H},D_{m,K}\right)$ are the moment of inertia and the damping coefficient matrices of the rotor; $T_{m}={\left[T_{m,H},T_{m,K}\right]}^{T}$ is the motor torque vector which is proportional to the motor current $I_{m}={\left[I_{m,H},I_{m,K}\right]}^{T}\;$. Namely:

(6) $$T_{m}=K_{m}I_{m}$$

where $K_{m}=\mathrm{diag}\left(K_{m,H},K_{m,K}\right)$ is the motor constant matrix.

In this paper, the driver built-in velocity servo control loop is activated. In other words, the built-in velocity controller accepts the velocity command ${\dot{\theta }}_{mc}={\left[{\dot{\theta }}_{mc,H},{\dot{\theta }}_{mc,K}\right]}^{T}$ from the admittance controller to be introduced in next subsection, and delivers the motor current $I_{m}={\left[I_{m,H},I_{m,K}\right]}^{T}$ such that ${\dot{\theta }}_{m}$ follows ${\dot{\theta }}_{mc}\;$. From the official manual of the motor manufacturer, the built-in velocity controller is equivalent to the following form:

(7) $$I_{m}(s)=C_{V2}(s)({\dot{\theta }}_{mc}(s)-C_{V1}(s){\dot{\theta }}_{m}(s))$$

Note that we present (7) in the Laplace domain and denote $I_{m}\left(s\right)$ and $\theta_{m}\left(s\right)$ as the Laplace transforms of $I_{m}\left(t\right)$ and $\theta_{m}\left(t\right)$, respectively. In addition, $C_{Vi}\left(s\right)=\mathrm{diag}\left(C_{Vi,H}\left(s\right),\;C_{Vi,K}\left(s\right)\right),\;i=1,\;2$ denote the feedback and feedforward equivalent velocity controllers, respectively.

**Figure 4:**
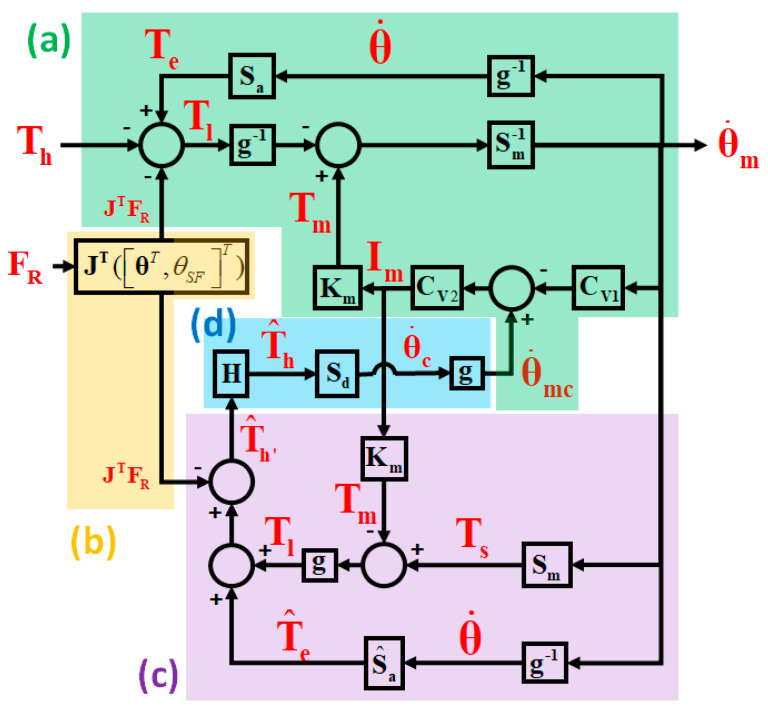
Block diagram of the single-leg exoskeleton control system; (**a**) integrated dynamics including motors, drivers and the exoskeleton mechanism; (**b**) ground reaction force (GRF) compensator; (**c**) admittance law; and (**d**) disturbance observer.

Combining (1) and (4)–(7), we can represent the dynamics of a single leg as the block diagram in Figure 4a. In addition, we define the blocks of the systems labeled $S_{a}$, ${\hat{S}}_{a}$, and $S_{m}$ in Figure 4 as follows:

(8) $$T_{e}=S_{a}\dot{\theta }=M(\theta )\ddot{\theta }+C(\theta ,\dot{\theta })\dot{\theta }+G(\theta )+B(\dot{\theta })$$

(9) $${\hat{T}}_{e}={\hat{S}}_{a}\dot{\theta }$$

(10) $$T_{s}=S_{m}{\dot{\theta }}_{m}=N_{m}{\ddot{\theta }}_{m}+D_{m}{\dot{\theta }}_{m}$$

where $S_{a}$ is a nonlinear operator that maps the joint velocity $\dot{\theta }$ to the right-hand side of (8). $S_{a}$ consists of *exact* and *unknown* system parameters in **M**(θ), $C\left(\theta ,\dot{\theta }\right)$, **G**(θ) and $B\left(\dot{\theta }\right)$, which means that $S_{a}$ includes the user’s lower limbs as a part of HES. The ${\hat{S}}_{a}$ of (9) is a nonlinear operator, which is the same as $S_{a}$ except that it consists of the nominal parameters in Table 1. Similarly, $S_{m}$ is a linear operator that maps the motor velocity ${\dot{\theta }}_{m}$ to the right-hand side of (10). $T_{e}={\left[T_{e,H},T_{e,K}\right]}^{T}$ and $T_{s}={\left[T_{s,H},T_{s,K}\right]}^{T}\;$are the equivalent joint torques from the movement of the exoskeleton and the torque exerting on the motor’s rotor, respectively. These definitions will simplify the derivation of the admittance control law and the robust stability analysis in the subsequent sections.

### 2.4. Disturbance Observer-Based Torque Estimator and Admittance Control

As shown in (1), $T_{h}$ is the user’s torque to HES, which indicates how the user would like to move the HES. Hence, we propose to estimate $T_{h}$ and treat it as the user’s motion intention. Then, the joint velocities are determined accordingly. Based on the dynamic model (1) and (4)–(7), or the block diagram of Figure 4a, we proposed in our previous research [22,23] a disturbance observer (DOB)-based torque estimator for estimating $T_{h}\;$. The proposed torque estimator is:

(11) $${\hat{T}}_{h}=H\left[{\hat{S}}_{a}\dot{\theta }+g\left(S_{m}{\dot{\theta }}_{m}-K_{m}I_{m}\right)-J^{T}({\left[\theta^{T},\theta_{SF}\right]}^{T})F_{R}\;\right]$$

Once the user’s torque has been estimated, it can be used to determine the desired joint angular velocity as shown in (12) and (13):

(12) $${\dot{\theta }}_{mc}=g{\dot{\theta }}_{c}=gS_{d}{\hat{T}}_{h}$$

(13) $$S_{d}={\left(N_{d}s+D_{d}\right)}^{-1}$$

where ${\dot{\theta }}_{c}$ and ${\dot{\theta }}_{mc}$ are the angular velocity command to the joint and the motor, respectively. $S_{d}$ is a first-order system with a torque input and an angular velocity output. Such a system is called *mechanical admittance* (or *admittance* for short). $S_{d}$ consists of two admittance parameters $N_{d}=\mathrm{diag}\left(N_{d,H},N_{d,K}\right)$ and $D_{d}=\mathrm{diag}\left(D_{d,H},D_{d,K}\right)\;$, representing the desired inertia and damping coefficients, respectively. Roughly speaking, if the torque estimation and the motor velocity servo control are accurate, i.e., $T_{h}\approx {\hat{T}}_{h}$ and $\dot{\theta }\approx {\dot{\theta }}_{c}\;$, then $S_{d}$ becomes the admittance of HES. Since **N_d_** and **D_d_** can be assigned arbitrarily, provided that $S_{d}$ is stable, we can use the admittance control law to change the admittance of HES to any predefined function $S_{d}\;$. If $S_{d}$ has a high gain (or **N_d_** and **D_d_** are small), then the joints can move faster with a small torque. As a result, the user walks faster and feels more energy-efficient. Rigorous analysis on the robust stability of the closed-loop system will be presented in the next section.

## 3. Robust Stability Analysis of Walking

Since HES contains a feedback loop in it (see Figure 4) and the torque estimator (11) is based on the nominal model, it is crucial to guarantee the closed-loop stability of HES in the presence of model uncertainties. In this section, we derive sufficient conditions for the robust stability of the closed-loop system in Figure 4. Due to the feedback of GRF to the torque estimator, the stability conditions in this paper are slightly different from those in [23], where the GRF is ignored. Because the closed-loop system includes nonlinear terms in $S_{a}$ and ${\hat{S}}_{a}\;$, the notion of finite-gain ${ℒ}_{2}$ stability is considered here. Readers can find the basic definitions regarding the ${ℒ}_{2}$-norm of a signal, ${ℒ}_{2}$-gain, and finite-gain ${ℒ}_{2}$ stability of a system in [25]. We conducted a stability analysis in Section 3.1, and present the admittance control system under the condition of precise velocity servo control in Section 3.2.

### 3.1. Robust Stability Analysis for the Closed-Loop System

Note that $S_{a}$ and ${\hat{S}}_{a}$ defined in (8) and (9) are nonlinear mappings from $\dot{\theta }$ to $T_{e}$ and ${\hat{T}}_{e}$, respectively. Due to the nonlinearity of $S_{a}$ and ${\hat{S}}_{a}$, the following analysis is derived in the time domain. Therefore, all the blocks in Figure 4 are regarded as input–output mappings in the time domain. Moreover, the blocks including $S_{m}$, $K_{m}$, **g**, $C_{V1}$, and $C_{V2}$ are all linear time-invariant (LTI) and diagonal. Therefore, the series connections of these blocks are commutable. To smooth the derivation, we define the following input–output mappings in (14)–(17) that will be used in the analysis. Notice **I** here is the identity matrix:

(14) $$\alpha_{1}=(I+g^{-2}S_{m}^{-1}S_{a}+S_{m}^{-1}K_{m}C_{V2}C_{V1}{)}^{-1}$$

(15) $$\alpha_{2}=S_{m}^{-1}K_{m}C_{V2}S_{d}$$

(16) $$\beta =I-g^{2}K_{m}C_{V2}S_{d}H{\left(I+g^{2}K_{m}C_{V2}S_{d}H\right)}^{-1}$$

(17) $$\gamma =S_{m}^{-1}(g^{-2}+K_{m}C_{V2}S_{d}H)$$

Based on Figure 4, we can derive the relationship from the user’s joint torque $T_{h}\;$and GRF $F_{R}$ to the joint velocity $\dot{\theta }\;$. The result is shown as (18), where $\nabla =S_{a}-{\hat{S}}_{a}$ is the model uncertainty. We define $T_{input}$ as the sum of $T_{h}$ and $\beta J^{T}F_{R}$, where β is defined in (16):

(18) $$\dot{\theta }={(\alpha_{1}^{-1}+\alpha_{2}H\nabla )}^{-1}\gamma (T_{h}+\beta J^{T}F_{R})={(\alpha_{1}^{-1}+\alpha_{2}H\nabla )}^{-1}\gamma T_{input}$$

It is reasonable to assume that **C_V1_** and **C_V2_** stabilize the velocity loop of the motor, and **C_V2_** is stable. Suppose that the desired admittance function **S_d_** and the filter **H** are finite-gain ${ℒ}_{2}$ stable. **g** and **K_m_** are the constant matrix. We assume that β , or equivalently ${\left(I+g^{2}K_{m}C_{V2}S_{d}H\right)}^{-1}$, is finite-gain ${ℒ}_{2}$ stable. Then, the ${ℒ}_{2}$-norm of the $T_{input}$ is bounded, provided that the ${ℒ}_{2}$-norms of $T_{h}\;$and $F_{R}$ are bounded. Under these conditions, the procedure for deriving robust stability in [23] can be directly applied to (18). Assuming that ∇ is finite-gain ${ℒ}_{2}$ stable and H∇≤a for some a>0 . Then, we follow the procedure in [23] and can reach the conclusion that the closed-loop system (18) is finite-gain ${ℒ}_{2}$ stable if (19) is satisfied:

(19) $$\left\|\alpha_{1}\alpha_{2}\right\|<1/a$$

We summarize the robust stability analysis as the following Theorem 1.

**Theorem** 1. *If the exoskeleton system in Figure 4 satisfies the following assumptions*:

1. ***C_V2_****, **S_d_**,and **H**are finite-gain*${ℒ}_{2}$*stable*;
2. $\alpha_{1}$*is stable*, *i.e.*, *the velocity control loop of the joint motor is stable*;
3. ${\left(I+g^{2}K_{m}C_{V2}S_{d}H\right)}^{-1}$*is stable*;
4. *The model uncertainty*∇*is finite-gain *${ℒ}_{2}$*stable and*‖H∇‖<a*for some*0<a<∞ ;
5. $\|\alpha_{1}\alpha_{2}\|<1/a$;

*then the exoskeleton system is finite-gain *${ℒ}_{2}$*stable*.

### 3.2. Admittance Control System under Precise Velocity Servo Control

The state-of-the-art motor servo control technologies allow **C_V1_** and **C_V2_** to achieve precise velocity tracking, i.e., ${\dot{\theta }}_{m}\approx {\dot{\theta }}_{mc}\;$. As is well known by control engineers, precise tracking control relies on high-gain controllers in the loop, i.e., $\|C_{V2}\|\gg 1\;$. In such a circumstance, we can get (20) from (16), which implies that GRF $F_{R}$ has no effect on the joint velocity $\dot{\theta }$ in (18).

(20) $$\beta =I-g^{2}K_{m}C_{V2}S_{d}H{\left(I+g^{2}K_{m}C_{V2}S_{d}H\right)}^{-1}\approx 0$$

Following the same arguments as in [23], we can also get the following conclusions:

1. The gain of the admittance function is inversely proportional to the size of the model uncertainty:
  (21) $$\left\|\alpha_{1}\alpha_{2}\right\|\approx \left\|S_{d}\right\|<1/a$$
2. If the identified model ${\hat{S}}_{a}$ is close to the actual one $S_{a}\;$, i.e., the uncertainty ∇ is negligible, then:
  (22) $$\dot{\theta }=S_{d}HT_{input}=S_{d}HT_{h}$$

Combing (21), (22) and the fact that the uncertainty is more manifest in the high-frequency band while the bandwidth of the human motion is relative low, we conclude that **H** should be a low-pass filter.

## 4. Experimental Results and Discussion

The accuracy and robustness of torque estimation under the condition of no GRF have been experimentally verified in our previous work [22,23]. In this paper, we aimed at investigating how the proposed admittance control law affects the user’s level ground walking. As a first step, we verify by experiments that the effects of GRF on the estimated user’s torque can be eliminated if GRF is measured by the module we devised in Section 2.1. Then, the experiments on the level ground assistive walking with different admittance parameters are conducted in Section 4.2 to show that the user’s gaits can be shaped in a desired way. In Section 4.3, we recruit three test subjects who are significantly different in their height and weight, and repeat the level ground assistive walking experiments with the exhaustive exploration of admittance parameters. To quantitatively analyze the experimental results, we define the normalized energy consumption index (NECI) and walking distance as the performance criteria. Based on these criteria, we show that the proposed admittance control law reduces the energy consumption of the user in level ground walking.

### 4.1. Elimination of the Influence of GRF

To verify that the module in Section 2.1 can effectively eliminate the influence of GRF, we dangled the exoskeleton on a rack with no user in it (i.e., $T_{h}=0\;$) and no contact with the ground as in Figure 1a. Then, we applied forces by hand on the FSRs installed at the feet to simulate the effects of GRF, and calculated its equivalent torque by $J^{T}\left({\left[\theta^{T},\theta_{SF}\right]}^{T}\right)F$, where F is force measured from the FSRs. From the torque estimator (11) we see that this equivalent torque should be cancelled out by the term ${\hat{S}}_{a}\dot{\theta }+g\left(S_{m}{\dot{\theta }}_{m}-K_{m}I_{m}\right)$ since $T_{h}=0$ in this case.

**Figure 5:**
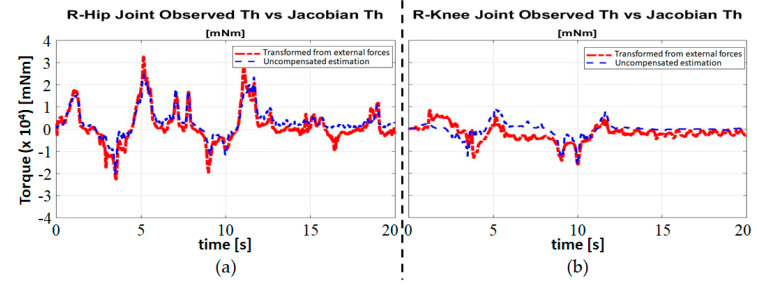
The uncompensated user’s joint torque estimation (red long dashed line) and the equivalent joint torque due to the external force (blue short dashed line): (**a**) right hip; and (**b**) right knee.

Figure 5 shows the experimental results of the right hip and right knee joints. The red long dashed line represents the term ${\hat{S}}_{a}\dot{\theta }+g\left(S_{m}{\dot{\theta }}_{m}-K_{m}I_{m}\right)$, which is called the *uncompensated user’s torque estimate* here because it is the user’s torque estimate without compensating for GRF. The blue short dashed line represents the equivalent joint torque due to the external force, $J^{T}\left({\left[\theta^{T},\theta_{SF}\right]}^{T}\right)F$. We can observe that they are very close but not exactly the same. This indicates that the effects of GRF can be properly compensated for, resulting in a sufficiently accurate user’s joint torque estimation during walking. However, the small residue in Figure 5 should be taken into account when admittance parameters are tuned. We will explore this issue in more detail in the next subsection.

### 4.2. Level Ground Walking Tests

In this subsection, we conducted experiments of level ground assistive walking with different admittance parameters to show that the user’s gait can be shaped by the admittance control law in a desired way. The photos of a healthy test subject wearing the exoskeleton and standing on the ground are shown in Figure 1b,c For safety reasons, the test subject was recommended to hold crutches during walking. The test subject was 170 cm tall, and weighed 77 kg, while the exoskeleton weighed 25.5 kg. During each experiment, the test subject was asked to walk freely in their most comfortable way for 20 s. We also asked the test subject to keep the inclination angle of the torso as small as possible, since a zero-inclination angle was implicitly assumed in the model (1). In the future, this issue will be resolved by measuring the inclination angle with an inertia measurement unit (IMU) installed at the lower back of the exoskeleton and compensating for it in the model-based torque estimator.

**Figure 6:**
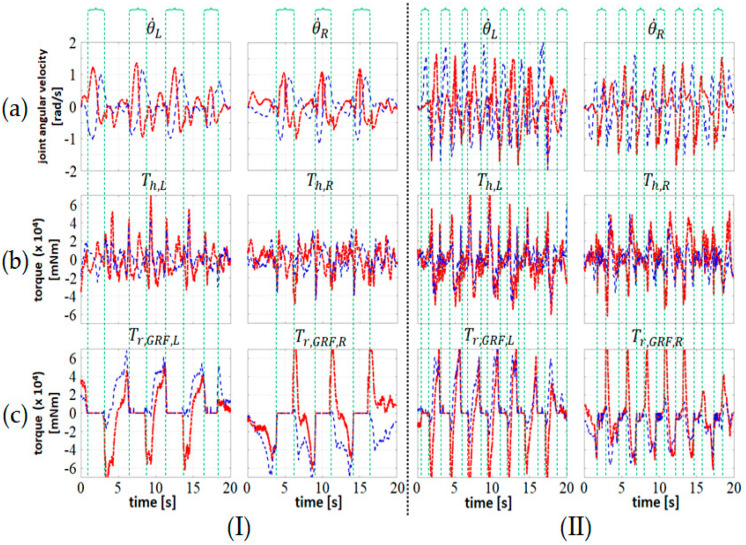
The joint-related information of the walking experiments under different admittance parameters. (**I**) $\left(N_{d,H}=N_{d,K},D_{d,H}=D_{d,K}\right)=\left(5000,\;5000\right)$; (**II**) $\left(N_{d,H}=N_{d,K},D_{d,H}=D_{d,K}\right)=\left(1000,\;3000\right)$; (**a**) the joint angular velocity $\dot{\theta }$; (**b**) the estimated user’s torque ${\hat{T}}_{h}$; and (**c**) the equivalent joint torque due to GRF. Red dot-dashed lines represent the hip joint and the blue dashed lines represent the knee joint. Subscripts **R** and **L** denote the right-leg and left-leg related data, respectively.

Because the imperfect cancellation of the equivalent joint torque due to GRF and coupling between two legs, the small residue shown in Figure 5 or the internal force between two hip joints may cause the vibration of the exoskeleton if it is amplified by a high-gain admittance function **S_d_**. To attenuate the vibration while providing sufficient assistive torques during walking, we chose an admittance function with large inertia and damping coefficients in the stance phase, and switched to an admittance function with small inertia and damping coefficients in the swing phase. After some trials, we chose $N_{d}=\mathrm{diag}\left(15,000,\;20,000\right)$ and $D_{d}=\mathrm{diag}\left(15,000,\;20,000\right)$ in the stance phase. In the swing phase, we used two sets of parameters which were $N_{d}=\mathrm{diag}\left(5000,\;5000\right)\;$, $D_{d}=\mathrm{diag}\left(5000,\;5000\right)$ as case (I), and $N_{d}=\mathrm{diag}\left(1000,\;1000\right)\;$, $D_{d}=\mathrm{diag}\left(3000,\;3000\right)\;$as case (II). The results are shown in Figure 6 and we can compare both cases to justify how the admittance parameters affect the gait.

Notice that it is easy to distinguish the stance phase from the swing phase, since GRF, or its equivalent joint torque, is zero during the swing phase (see Figure 6c). Comparing the two sets of admittance parameters, we see that case (I) is assigned a larger inertia and damping coefficients with both joints. Therefore, the subject is expected to experience a heavier load on his lower limb and experience more difficulty moving than the case (II). From Figure 6 we see that in both cases, the user’s torques have similar magnitudes; however, the gait cycle time of “lighter” parameters (i.e., case (II)) is much shorter than that using “heavier” parameters. In addition, the walking distances in 20 s of case (I) and (II) are 205 cm and 580 cm, respectively. These observations show that the test subject attempts to walk faster while keep the same level of joint torques when a set of smaller admittance parameters is applied.

Now, we demonstrated that the proposed admittance control method can shape the user’s gait by tuning the admittance parameters. In the next subsection, we will define physically insightful performance indices to quantitatively evaluate the effects of different admittance parameters

### 4.3. Performance Indices Based on Collected Data

During the level ground assistive walking experiments, data including the estimated test subject’s joint torque ${\hat{T}}_{h}\;$, and the joint angular velocity $\dot{\theta }$ are collected with a sampling time of 20 milliseconds. For easy reference in the subsequent derivation, we used the subscripts *H*, *K*, *R* and *L* to denote the data associated with the hip, knee, right leg and left leg, respectively, while the index in parentheses represents the time sample. Supposing that *N* samples of data are collected during one experiment, then the *normalized energy consumption index* (NECI) associated with that experiment is defined as:

(23) $$NECI=\frac{1}{M}\sum_{\begin{array}{l} i=H,K\\ j=R,L \end{array}}\left(\frac{\sum_{k=0}^{N-1}{\hat{T}}_{h,i,j}(k){\dot{\theta }}_{i,j}(k)}{\sum_{k=0}^{N-1}\left|{\dot{\theta }}_{i,j}(k)\right|}\right)$$

where *M* is the test subject’s weight. The numerator of (23) is the total energy exerted from the test subject to each joint of HES, while the denominator is the total angular distance traveled by that joint. The ratio represents the energy consumed by the test subject on a joint when the joint moves by unit angular distance; therefore, it is independent of the walking speed. For example, if the walking speed is higher, the test subject must consume more energy; however, the angular distance is also longer and the ratio in (23) keeps the same value. Then, the NECI is defined as the sum of the ratios for the four joints (the hip and knee joints of both legs) and is normalized by the weight of the test subject. Thus, NECI represents the intrinsic biomechanical property of the test subject’s lower limbs and its value should be comparable between the different test subjects under different walking speed.

The advantages of NECI are evident. Firstly, NECI is calculated based on the data collected from the exoskeleton (${\hat{T}}_{h}$ and $\dot{\theta }\;$). No extra sensors or devices are required. Secondly, NECI is independent of the data length and the test subject since it is normalized with respect to the angular distance and the weight of the test subject. Hence, NECI only reflects the differences resulting from the admittance parameters. The higher the NECI is, the more energy the test subject consumes in walking.

Another performance index we used in this paper was the walking distance. The duration of each experiment was fixed to 20 s. After the end of an experiment, the walking distance was measured and recorded. Since the time duration was fixed, a longer walking distance means a faster walking speed. Therefore, we can compare the effects of the admittance parameters on the walking speed.

#### 4.3.1. Performance Indices of the Level Ground Walking Tests

**Figure 7:**
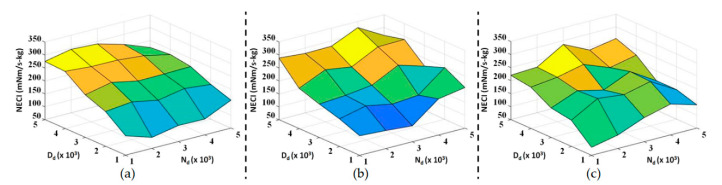
Three-dimensional plot of *normalized energy consumption index* (NECI) for a series of experiments as **N_d_** and **D_d_** vary from 1000 to 5000: (**a**) Subject 1; (**b**) Subject 2; and (**c**) Subject 3.

**Figure 8:**
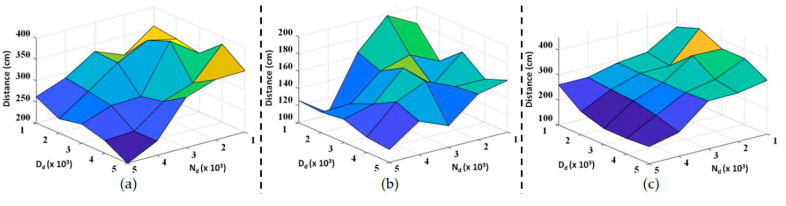
Three-dimensional plot of walking distance for a series of experiments as **N_d_** and **D_d_** vary from 1000 to 5000: (**a**) Subject 1; (**b**) Subject 2; and (**c**) Subject 3.

**Figure 9:**
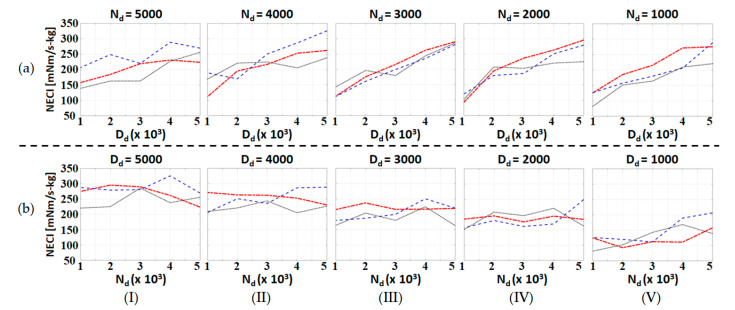
Plot of *NECI* for a series of experiments with different admittance parameters: (**a**) **N_d_** is fixed and **D_d_** varies; and (**b**) **D_d_** is fixed and **N_d_** varies. The red dot-dashed line, blue dashed line and the black dotted line represent the data of Subject 1, Subject 2 and Subject 3, respectively.

**Figure 10:**
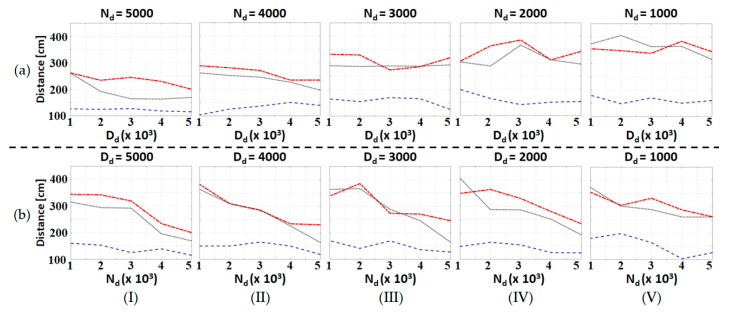
Plot of THE walking distance for a series of experiments with different admittance parameters: (**a**) **N_d_** is fixed and **D_d_** varies; and (**b**) **D_d_** is fixed and **N_d_** varies. The red dot-dashed line, blue dashed line and the black dotted line represent the data of Subject 1, Subject 2 and Subject 3, respectively.

In this subsection, we repeated the level ground assistive walking experiments with three different test subjects and exhaustive combinations of admittance parameters. The basic information of the test subjects is listed in Table 2, and both **N_d_** and **D_d_** vary from diag(1000, 1000) to diag(5000, 5000). NECI and the walking distance of all the experimental data are shown in Figure 7a,c and Figure 8a–c, respectively. Moreover, the dependence of these performance indices with respect to one admittance parameter when the other is fixed is shown in Figure 9 and Figure 10. Notice that all the experiments use the same nominal parameters shown in Table 1 for ${\hat{S}}_{a}\;$.

According to Figure 7, NECI decreases as **D_d_** decreases, but there are no obvious differences when **N_d_** is varying. This trend can be seen more clearly from Figure 9. When we fix **N_d_** and adjust **D_d_**, all the curves in Figure 9a show the same downward trend towards a smaller **D_d_**. On the other hand, NECI is hardly affected by **N_d_** since the curves in Figure 9b show no obvious trend with respect to **N_d_**.; however, the mean value of NECI for the different **N_d_** becomes smaller when **D_d_** is fixed at a smaller value. As the result, we conclude that the user of the exoskeleton consumes less energy in walking when we choose a smaller damping coefficient, **D_d_**.

Figure 8 shows that the walking distance increases as **N_d_** decreases. This result can be seen more clearly from Figure 10b. Besides, **D_d_** seems to have no definite influence on the walking distance as shown in Figure 10a.

To summarize, we showed that the proposed admittance control law can reduce the energy consumption of the user in the level ground walking by decreasing the damping coefficient **D_d_** of the desired admittance function. We can also increase the walking speed by decreasing the inertia **N_d_** of the desired admittance function. However, one question remains unsolved. Since all the comparisons of the performance indices are under the condition of using the exoskeleton for assistive walking, one might be curious about the performance indices for a healthy person walking without the exoskeleton. From the experimental data, we see that the walking speeds of all three test subjects are lower than the normal value, which is between 1.04 m/s and 1.50 m/s for younger pedestrians [26]. The possible reason is that the first order admittance function (13) does not coincide with the inherent human joint admittance. Therefore, the test subjects feel unnatural when the exoskeleton is involved in the assistive walking. Consequently, the test subjects tend to walk cautiously and slowly to assure their stability. The possible solutions are (1) asking the test subject to practice more and get used to the assistance from the exoskeleton, or (2) modifying the admittance functions. However, finding an energy-efficient and “natural” admittance function is an open question and is beyond the scope of this paper. We will explore this topic in future research.

Evaluating NECI for people walking without the exoskeleton is more challenging since it requires specialized equipment to measure or estimate the joint’s angular velocities and joint torques. Instead of collecting the experimental data of walking without the exoskeleton, we present the data obtained from an open source software, and compared them with the results of this subsection.

#### 4.3.2. NECI Value for a Healthy Subject without the Exoskeleton

**Figure 11:**
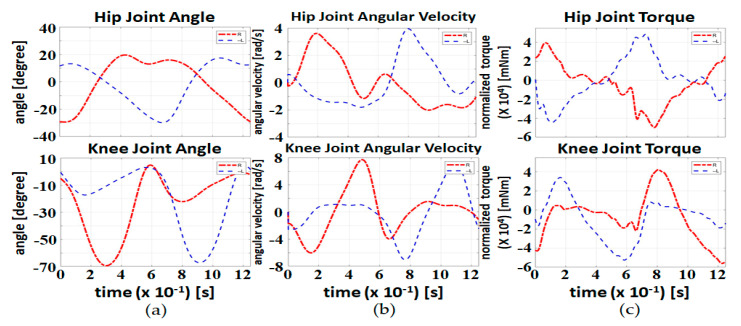
Joint data of one gait cycle obtained from the open source software: (**a**) joint angle; (**b**) joint angular velocity; (**c**) normalized joint torque. The red dot-dashed line and blue dashed line represent the data of the right leg and the left leg, respectively.

Figure 11 presents one gait cycle data of a healthy subject walking without the exoskeleton. The data are obtained from OpenSim 4.0 [27,28], an open source software for biomechanical modeling, the simulation and analysis developed by Stanford University. OpenSim builds a universal human musculoskeletal model based on muscle morphological parameters. Users can scale the universal model with respect to the body features of a test subject (high, weight, etc.) and build a personalized model for the test subject. Then, inverse kinematics is applied to match the personalized model to the recorded movement of the test subject. Then, the residual reduction algorithm (RRA) and the computed muscle control (CMC) algorithm combine the external forces (i.e., GRF) and generate muscle forces and joint torques.

We adopted the core model “gait10dof18musc” provided by the official OpenSim to produce the data in Figure 11. The model includes the trunk, pelvis and leg segments with 10 degrees of freedom and 18 muscles. The test subject of the model weighs 72.6 kg and is 180 cm tall. The sampling rate of the data is 1000 Hz. We used the data in Figure 11 as a reference for walking without the exoskeleton.

The NECI value of the data is 174.54 mNm/s-kg. Note that this value is in the same numerical range of the NECI values presented in Section 4.3.1, implying that the NECI is comparable among different test subjects with different walking speeds. Therefore, the NECI value of walking without the exoskeleton is used as the benchmark to compare with the experimental results in Section 4.3.1. Then, we can realize the differences in energy consumption of a healthy person walking with and without the exoskeleton. For the ease of comparison, we calculate the ratio of the NECI values in Section 4.3.1 to the benchmark and show the results in Table 3.

There are three numbers in each field of Table 3, indicating the aforementioned ratios for the three test subjects. If the ratio is larger than 1, the test subject consumes more energy than the benchmark. On the contrary, if the ratio is smaller than 1, it means that assistive walking by the admittance control law saves the energy of the test subject. For Subject 1, 2, and 3, they can save up to 47%, 36% and 53% of the energy with respect to the benchmark, respectively. We can also notice that for some particular sets of admittance parameters, the ratios are close to 1, which means that the energy consumption is roughly equal to the benchmark. Comments from the test subjects point out that they feel more natural in assistive walking when these sets of admittance parameters are applied. This reveals some clues for the future design of the admittance function that makes the exoskeleton nearly “transparent” to the user.

## 5. Conclusions

In this paper, we designed and implemented an admittance control system of the exoskeleton that makes energy-efficient assistive walking possible. The control system first compensates for the effects of GRF and then accurately estimates the torque from the user to the human-exoskeleton system (HES). In addition, the control system shapes the user’s gait by tuning the admittance function which has the input from the estimated user’s torque and the output to the joint angular velocity. Rigorous theoretical analysis on the robust stability of the closed-loop system is performed. Then, we proposed performance indices such as NECI and walking distance to evaluate the effects of each individual admittance parameter on the level ground assistive walking. Comparing the NECI value for a healthy person walking without exoskeleton, we found that energy-saving walking can be achieved.

According to the results of this paper, we conclude the advantages of the proposed methods:

1. The proposed admittance control system and GRF compensator can accurately estimate the user’s exerting torque to identify the walking intention and conduct assistive walking. No biological sensors are needed. In addition, the admittance is online adjustable which can shape the admittance of HES to adapt the gaits to different walking conditions in real time.
2. We proposed performance indices such as NECI and walking distance that can be easily calculated from the data collected by the exoskeleton. No extra devices or evaluating procedures are required. Moreover, these indices give physical insights of the performance in the perspectives of the user’s energy consumption and walking speed.
3. By comparing the results of the benchmark with the experimental data in Section 4.3.1, we claim that the proposed admittance control system is able to save the energy of the user during level ground walking. More precisely, the damping coefficient **D_d_** of the admittance function regulates the energy consumption whereas the inertia **N_d_** affects the walking speed.

In the future, we will establish an admittance function that allows the user to walk in a natural and more energy-efficient way. Meanwhile, the walking speed can be as high as the user expects. In addition, we would like to experimentally compare NECI values with other commonly used performance evaluation methods such as those based on sEMG or metabolic cost to verify the effectiveness of NECI.

## Figures and Tables

**Table 1:** Identified parameter values of the right leg.

Parameters	Units	Value
$F_{TS}$	mNm	8342.99 (positive direction)
		8873.57 (negative direction)
$M_{S}L_{SC}G$	mNm	3785.07
$I_{S}+M_{S}L_{SC}^{2}$	mNm/(1/s^2^)	2804.41
$B_{TS}$	mNm/(1/s)	4915.77
$M_{S}L_{T}L_{SC}$	mNm/((1/s)^2^)	162.22
$F_{BT}$	mNm	9321.29 (positive direction)
		9475.27 (negative direction)
$(M_{T}L_{TC}+M_{S}L_{T})G$	mNm	18260.22
$\left(I_{T}+M_{T}L_{TC}^{2}+M_{S}L_{T}^{2}\right)$	mNm/(1/s^2^)	3000
$B_{BT}$	mNm/(1/s^2^)	5000

**Table 2:** Basic information of the test subjects.

Test Subject	Gender	Age	Height (cm)	Weight (kg)
**Subject 1**	Male	29	170	77
**Subject 2**	Male	22	180	60
**Subject 3**	Male	30	164	85
**Exoskeleton**				25.5

**Table 3:** Ratios of the NECI values for level ground walking with different admittance parameters with respect to the benchmark. The numbers without parentheses, with parentheses and with brackets are the results of Subject 1, Subject 2 and Subject 3, respectively.

D_d_\N_d_	5000	4000	3000	2000	1000
**5000**	1.28 (1.54) [1.47]	1.50 (1.87) [1.36]	1.66 (1.61) [1.64]	1.69 (1.60) [1.29]	1.58 (1.65) [1.26]
**4000**	1.32 (1.65) [1.30]	1.45 (1.64) [1.17]	1.50 (1.35) [1.40]	1.51 (1.44) [1.26]	1.55 (1.18) [1.20]
**3000**	1.25 (1.26) [0.93]	1.24 (1.43) [1.28]	1.24 (1.14) [1.03]	1.36 (1.07) [1.17]	1.23 (1.03) [0.94]
**2000**	1.05 (1.42) [0.93]	1.12 (0.97) [1.26]	1.01 (0.92) [1.12]	1.12 (1.03) [1.19]	1.06 (0.90) [0.86]
**1000**	0.90 (1.18) [0.80]	0.63 (1.08) [0.95]	0.64 (0.64) [0.82]	0.53 (0.69) [0.59]	0.72 (0.72) [0.47]
